# Feasibility of Increasing Childhood Outdoor Play and Decreasing Television Viewing Through a Family-Based Intervention in WIC, New York State, 2007-2008

**Published:** 2011-04-15

**Authors:** Kirsten K. Davison, Lynn S. Edmunds, Laurie M. Young, Vanessa S. Sarfoh, Brett A. Wyker, Jackson P. Sekhobo

**Affiliations:** University at Albany, State University of New York, Albany, New York; Evaluation and Analysis Unit, Bureau of Administration and Evaluation, Division of Nutrition, New York State Department of Health; University at Albany, State University of New York, Albany, New York; University at Albany, State University of New York, Albany, New York; Division of Nutrition, New York State Department of Health, Albany, New York; Division of Nutrition, New York State Department of Health, Albany, New York

## Abstract

**Introduction:**

Active Families is a program developed to increase outdoor play and decrease television viewing among preschool-aged children enrolled in the Special Supplemental Nutrition Program for Women, Infants, and Children (WIC). Our objective was to assess its feasibility and efficacy.

**Methods:**

We implemented Active Families in a large WIC clinic in New York State for 1 year. To this end, we incorporated into WIC nutrition counseling sessions a community resource guide with maps showing recreational venues. Outcome measures were children's television viewing and time playing outdoors and parents' behaviors (television viewing, physical activity), self-efficacy to influence children's behaviors, and parenting practices specific to television viewing. We used a nonpaired pretest and posttest design to evaluate the intervention, drawing on comparison data from 3 matched WIC agencies.

**Results:**

Compared with the children at baseline, the children at follow-up were more likely to watch television less than 2 hours per day and play outdoors for at least 60 minutes per day. Additionally, parents reported higher self-efficacy to limit children's television viewing and were more likely to meet physical activity recommendations and watch television less than 2 hours per day.

**Conclusion:**

Results suggest that it is feasible to foster increased outdoor play and reduced television viewing among WIC-enrolled children by incorporating a community resource guide into WIC nutrition counseling sessions. Future research should test the intervention with a stronger evaluation design in multiple settings, with more diverse WIC populations, and by using more objective outcome measures of child behaviors.

## Introduction

The prevalence of obesity is increasing among children in the United States, particularly among low-income children (1). In 2003, the prevalence of obesity among young children living in low-income families was almost 15% (2) and in New York State exceeded 16% (3). Although obesity prevention programs targeting low-income preschool-aged children are needed, few such programs have been implemented (4).

The Special Supplemental Nutrition Program for Women, Infants, and Children (WIC) presents an ideal opportunity for health promotion efforts that target preschoolers and their families. WIC provides direct contact with parents, who are fundamental in shaping children's dietary and physical activity (PA) behaviors (5). Fit WIC is a WIC-based obesity prevention program (6). In New York State, Fit WIC incorporates obesity prevention into the re-education of WIC staff. The program provides training and resources for clinic staff in how to incorporate PA into nutrition assessment and education, offers WIC staff opportunities for wellness at work, and encourages staff to model healthy behaviors.

From 2007 through 2008, the New York State WIC program implemented a pilot project called Active Families to enhance the Fit WIC initiative. Given that at least 50% of preschoolers do not meet recommended levels of PA (7) and television viewing (8,9), Active Families sought to increase the time that children spend playing outdoors (10) and decrease the time they spend watching television (8). Outdoor play was a target behavior because it is consistently linked with higher PA among young children (11-13). Furthermore, it is challenging to quantify PA in preschool children because of the intermittent nature of their activity patterns (14). To achieve the goals of increasing outdoor play and reducing television viewing, Active Families addressed community-based barriers to children's PA and outdoor play (11,15) by integrating into WIC counseling sessions a community resource guide linking families with local resources for PA. The specific objective of our study was to assess the feasibility and efficacy of the Active Families program.

## Methods

### Study design and setting

We used a pre-post (nonpaired) quasiexperimental design with a nonequivalent comparison group. We implemented the Active Families program in a WIC clinic, in a metropolitan area in central New York State, from August 2007 through August 2008. We selected this clinic as the intervention site because it has a large and diverse client base and the staff had previously participated in Fit WIC training. The institutional review boards of the New York State Department of Health and the University at Albany, State University of New York, approved the study protocol.

We collected baseline data from 422 families at the target clinic during June, July, and August in 2007 and follow-up data from 442 families during July and August in 2008, representing approximately one-third of families with enrolled children during each time period. It was not possible to link the baseline and follow-up data because of budgetary, time, and logistical constraints associated with working in a community setting. Participants therefore reflect a sample of the clinic population at each time point. During both assessment periods, parents (defined in this article as biological parents or other home-based caregivers) of children aged 2 to 5 years completed a self-report survey, with reference to their oldest child enrolled in WIC, in the clinic waiting room. A trained research assistant provided assistance as necessary. Surveys were available in English and Spanish, although most parents completed the survey in English.

The survey measured the primary outcomes of interest and theory-based mechanisms (16,17) expected to explain intervention effects: 1) demographic factors, including child and parent age and sex, parent race/ethnicity, and parent education; 2) child television viewing, including hours per day the child watched television on a typical day and the presence of a television in the child’s bedroom; 3) child outdoor play or the time the child spent playing outdoors on a typical day, during the morning, afternoon, and early evening (18); 4) parent behaviors and parenting practices including hours per day the parent watched television, days per week the parent participated in at least 30 minutes of moderate PA or 20 minutes of vigorous PA, and whether the parent limited the child’s television viewing to less than 2 hours per day; and 5) parent self-efficacy to reduce the child’s television viewing time and encourage the child to be physically active. We modeled survey questions after previous statewide WIC surveys and validated surveys (18). The follow-up survey included process-related questions to examine whether parents received the guide, how many copies they received, whether they read the guide, and how they used the guide.

We drew comparison data from 3 matched WIC agencies in upstate New York. Eligibility criteria were 1) the agency had previously participated in Fit WIC training, 2) the agency was in an area geographically similar to the target site, and 3) the agency served a demographic population similar to the target site. At the comparison clinics, parents of children aged 2 to 5 years completed a survey during May, June, and July in 2008 (n = 458) as part of the Fit WIC evaluation (items matched those from the Active Families survey). This timeframe matched that of the follow-up assessment at the target clinic. Parents at the comparison sites (n = 398) also completed the survey during September, October, and November in 2006. Although the timing of this survey does not match that of the baseline assessment for the intervention site, we reviewed the earlier sets of data from the target and comparison sites for preexisting differences in the outcome variables. With 1 exception, we identified no differences that could bias results in favor of the target site. The exception was children's outdoor play; children from the target site were more active at baseline than were their counterparts at the comparison sites.

### Program description

The fundamental component of the Active Families program was a community resource guide linking families with local resources for PA. The guide included an extensive list of outdoor recreation venues, such as parks and playgrounds, with maps identifying the specific location of each venue along with information on hours of operation, contact details, costs, and available facilities. A calendar of community events, updated every 2 to 3 months, was included as an insert in the back of the guide to encourage outdoor family recreation (eg, local family festivals). We developed winter and summer versions of the guide. The winter guide outlined opportunities for indoor as well as outdoor recreation.

Before program implementation, we conducted 2 focus groups, with 5 and 8 people per group, to get feedback from parents on the usefulness of the guide, other places in their community for active recreation, and the topics that should be included in the guide. The first author (K.K.D.) and a public health dietitian experienced in moderating focus groups conducted each focus group. Based on parent feedback, we incorporated additional content in the guide, including information on the benefits of increasing children's PA and reducing television viewing, suggestions for non–screen-based indoor activities, where to find free or low-cost winter clothing, and responses to frequently asked questions that emerged during the focus groups.

### Program implementation

In July 2007, the authors (K.K.D., L.S.E., L.M.Y.) trained the clinic staff to use the guide as a tool during counseling sessions. In particular, we encouraged counselors to 1) review the guide with parents, 2) point out the goals of the program and the benefits of increasing PA and reducing television viewing, 3) show parents the maps and help them locate their home, and 4) bring their attention to the calendar of local events at the back of the guide. We repeated the training session midway through the year for any new staff.

From August 2007 through August 2008, WIC counselors at the target site distributed the community guide to parents of children aged 18 months or older during WIC counseling sessions. Guides were also available in the clinic waiting room. During the intervention, families received up to 4 copies of the guide. To ensure the program was implemented as intended, we conducted periodic site visits and observed the extent to which counselors used the guides during counseling sessions and whether guides were available in the counseling areas and waiting room.

### Statistical analysis

To facilitate data analysis and interpretation, we dichotomized the outcome variables. This structure allowed us to use the same analytic method for all the primary analyses. We coded child television viewing and PA to reflect whether the child met recommendations, including less than 2 hours per day of television viewing (8) and 60 minutes or more per day of playing outdoors, which we used as a surrogate for 60 minutes of PA (10). Similarly, we coded parent PA to reflect whether parents met recommended levels of PA (19). We coded questions specific to parent self-efficacy in reducing child television viewing and increasing child PA such that parents who strongly agreed or agreed with the statement "I am confident in my ability to reduce my child's television viewing time" or "I am confident in my ability to encourage my child to be physically active" were classified as having high self-efficacy. Those who indicated that they did not know, disagreed, or strongly disagreed were coded as having low self-efficacy. We coded race/ethnicity as white, black, Hispanic, or other/multiracial and parent education as some high school or less, high school graduate or equivalent, or at least some college.

In preliminary analyses, we examined differences in sample characteristics for the target (baseline and follow-up) and comparison sites using χ^2^ analysis or *t* tests, as appropriate. In the primary analyses, we assessed differences in outcome variables at baseline versus follow-up at the target site and at follow-up for the target versus comparison sites by using logistic regression analysis. We conducted all analyses using SAS version 9.2 (SAS Institute, Inc, Cary, North Carolina) and controlled for differences in parent characteristics identified in the preliminary analyses. Significance was established at *P* < .05.

## Results

### Sample characteristics

Significant baseline versus follow-up differences at the target site were observed for parent race/ethnicity and education (Table 1). Similarly, significant differences at the target site versus comparison sites at follow-up were identified for race/ethnicity and parent education. We observed no differences in the age or sex of children or the age of parents.

Across intervention and comparison sites and regardless of timing of the assessment, the majority of children watched less than 2 hours of television each day and played outdoors for at least 60 minutes each day (Table 2). The majority of parents watched television for more than 2 hours each day and were confident that they could limit their child's television viewing. Approximately half of parents met PA recommendations. Finally, approximately one-third of parents reported limiting children's television, and approximately half of children had a television in their bedroom.

### Program evaluation

Parents at follow-up compared with baseline were significantly more likely to report that 1) their child watched less than 2 hours of television per day, 2) they themselves watched television less than 2 hours per day, and 3) they felt confident they could limit their child's television viewing (Table 3). No significant group differences were identified for television in the child's bedroom and limits parents placed on the child's television viewing. For the PA outcomes, compared with baseline, parents at follow-up were significantly more likely to report sufficient PA to meet recommendations and that their children played outside for at least 60 minutes per day.

Compared with parents from the comparison clinics, parents from the target clinic were significantly more likely to report that they 1) watched television less than 2 hours per day, 2) were confident in their ability to limit their child's television viewing, and 3) limited their child's television viewing to less than 2 hours per day. With regard to PA, parents at the target site at follow-up were more likely to report that their children played outdoors for at least 60 minutes per day than were parents at comparison clinics.

### Process evaluation

Most of the parents who recalled receiving the guide (n = 86) reported that they read the guide (n = 62). The most frequently used section of the guide was the list of community events; approximately half of these parents indicated that they used this information. In addition, approximately 1 in 3 parents reported that they used the guide to be more active themselves, to help their child to be active, or to reduce their child's television viewing time. Parents also reported that they used the maps in the guide to find places to take their child (35%) and to find winter clothing for their child (10%). In terms of specific venues visited, 60% to 80% of parents who used the guide indicated that they visited parks, playgrounds, swimming pools, fairs, and festivals listed in the guide.

## Discussion

Our results suggest that building on existing nutrition counseling services in the WIC program by incorporating a community resource guide can increase the number of children meeting PA and television viewing recommendations. In 3 of 4 comparisons, the intervention was associated with an increased proportion of children playing outdoors for at least 60 minutes or watching television for less than 2 hours per day. Furthermore, in most instances we observed anticipated differences in potential mechanisms of effect (16,17), including parents' own behaviors and their confidence in their ability to influence their child's behavior. Results from this study are consistent with previous obesity prevention programs implemented in a WIC setting (20,21) and research showing that enhanced access to places for PA combined with information about possible activities is effective in increasing levels of PA (22).

To our knowledge, this is the first study to evaluate a community resource guide in a WIC setting to improve parent and child outcomes specific to PA and television viewing. Although we were unable to use a rigorous experimental design, the consistency of results supports the integrity of the observed intervention effect. Half of the comparisons were significant and in the anticipated direction. Additionally, no effects were in the opposite direction to those expected. Social desirability bias is a concern when relying on self-reported data, but it is unlikely that such a bias entirely explains the results because we would expect a similar bias in the comparison sample. It is also unlikely that preexisting group differences in the outcome variables are responsible for our findings. We identified few preexisting group differences between the target and comparison sites that would bias the results in favor of the intervention group. One exception is children's outdoor play. At baseline, parents at the target site reported higher scores for children's outdoor play than did parents at the comparison sites. Although this difference could indicate that children at the target site were more active overall at baseline, it more likely reflects a seasonal effect on PA (23) because baseline data were collected in summer for the target site and in fall for the comparison sites. As a result, it is difficult to interpret site differences in outdoor play for the target versus comparison sites.

We did not observe intervention effects for "television in the child's bedroom." Approximately 50% of children had a television in their bedroom, which is linked with more hours watching television (24) and is contrary to American Academy of Pediatrics recommendations (8). The common presence of a television in children's bedrooms could reflect sleeping arrangements in low-income families. For example, informal observations suggest that children in low-income families may be more likely to sleep in shared or communal areas where a television is often located. If this is the case, it has implications for how we define and measure the presence of a television in the child's "bedroom" and the associated intervention strategies. Unfortunately, although a substantial proportion of children across all income levels have a television in their bedroom, few interventions with known efficacy directly address this practice. Additional formative research is needed to understand factors that lead parents to allow a television in the area where children sleep and why it might be difficult to later reverse this decision.

This study has several limitations. Given the programmatic, community-based context in which we implemented the study and the limited funds available, it was not feasible to randomly assign clinics to a condition or to follow clients over time. The samples that we surveyed at baseline and follow-up, however, were representative of the clinic's population. Therefore, the results represent a site-level change in the outcomes of interest. Moreover, we distributed follow-up surveys at the same time of the year as baseline surveys to increase the likelihood that those surveyed at baseline would participate in the follow-up assessment (ie, WIC visits occur midway through a 6-month recertification period). Still, the lack of a randomized control group presents several threats to the internal validity of the study. Findings are also limited by the use of self-report measures of parenting practices and parent and child television viewing and PA behaviors. Finally, results are generally limited to English-speaking WIC-enrolled families.

Despite these limitations, results provide preliminary evidence for the feasibility and efficacy of the Active Families program and suggest that incorporating a community resource guide to promote PA into WIC counseling sessions is a strategy worthy of further investigation. Future research could expand on this initial work by using a more rigorous design with multiple WIC sites, using more objective outcome measures, and quantifying the resources necessary to develop resource guides on a larger scale.

## Acknowledgments

This study was funded by the US Department of Agriculture, Food and Nutrition Services, under a WIC Special Projects Grant to the New York State Department of Health. We thank Mark Giddings of the Division of Nutrition, New York State Department of Health, for his geographic information system and mapping expertise and technical assistance with development of the community resource guide.

## Figures and Tables

**Table 1:** Characteristics of Participants in the WIC Active Families Target and Comparison Sites, New York State, 2007-2008

**Characteristic**	**Target Site**		***P* Value**	**Comparison Sites, n = 458**	***P* Value^a^**

	**Baseline, n = 422**	**Follow-Up, n = 442**			
**Children**					
**Age, mean (SD), mo**	41.0 (10.5)	41.1 (10.5)	.84	40.5 (10.2)	.40
**Girls, %**	50.4	52.7	.50	49.8	.38
**Parents**					
**Race/ethnicity, %**					
White	33.3	30.4	.01	43.1	<.001
Black	38.3	47.2		46.2	
Hispanic	15.4	14.8		6.2	
Other/multiracial	13.0	7.5		4.4	
**Education, %**					
Some high school or less	37.9	38.5	.04	23.8	<.001
High school graduate or equivalent	27.3	33.7		45.7	
At least some college	34.8	27.8		30.5	
**Age, mean (SD), y**	28.8 (7.9)	29.4 (8.9)	.26	29.8 (7.3)	.45

Abbreviations: WIC, Special Supplemental Nutrition Program for Women, Infants, and Children; SD, standard deviation.

a Values indicate comparison sites vs follow-up for the target site. Data collection for the 2 groups corresponded in time period.*χ* ^2^ tests were used for categorical variables and *t* tests for continuous variables.

**Table 2:** Television Viewing and Physical Activity Outcomes for Participants in the WIC Active Families Target and Comparison Sites, New York State, 2007-2008

**Outcome**	**Target Site**		**Comparison Sites,** a ** %, n = 458**

	**Baseline, %, n = 422**	**Follow-Up, %, n = 442**	
**Television viewing**			
Child watches <2 h/d	59	66	67
Parent watches <2 h/d	25	44	30
Parent confident can limit child's television	70	90	78
Child does not have television in bedroom	51	51	41
Parent limits television to <2 h/d	30	39	26
**Physical activity (PA)**			
Child plays outdoors ≥60 min/d	74	83	67
Parent meets PA recommendations^b^	50	57	57
Parent confident can encourage PA	92	95	95

Abbreviation: WIC, Special Supplemental Nutrition Program for Women, Infants, and Children.

a Comparison site data collection corresponded in time period to target site follow-up data collection.

b 150 min/wk moderate-intensity or 75 min/wk vigorous-intensity aerobic PA (19).

**Table 3:** Odds of Television Viewing and Physical Activity Outcomes for Participants in the WIC Active Families Target and Comparison Sites, New York State, 2007-2008

**Outcome**	**Target Site at Follow-Up vs Baseline (Reference)**		**Target Site at Follow-Up vs Comparison Sites** a ** (Reference)**	

	**Adjusted OR** b **(95% CI)**	***P*** ** Value**	**Adjusted OR** b **(95% CI)**	***P*** ** Value**
**Television viewing**				
Child watches <2 h/d	1.40 (1.05-1.86)	.02	1.15 (0.83-1.57)	.39
Parent watches <2 h/d	2.79 (2.03-3.83)	<.001	2.37 (1.69-3.33)	<.001
Parent confident can limit child's television	4.14 (2.81-6.11)	<.001	3.32 (2.17-5.07)	<.001
Child does not have television in bedroom	1.08 (0.83-1.43)	.54	0.81 (0.60-1.09)	.17
Parent limits television to <2 h/d	1.25 (0.93-1.67)	.14	1.98 (1.43-2.75)	<.001
**Physical activity (PA)**				
Child plays outdoors ≥60 min/d	1.68 (1.19-2.37)	.003	2.79 (1.94-4.02)	<.001
Parent meets PA recommendations^c^	1.32 (1.01-1.73)	.04	1.20 (0.89-1.62)	.22
Parent confident can encourage PA	1.67 (0.95-2.95)	.07	1.61 (0.82-3.16)	.16

Abbreviations: WIC, Special Supplemental Nutrition Program for Women, Infants, and Children; OR, odds ratio.

a Comparison site data collection corresponded in time period to target site follow-up data collection.

b All ORs were adjusted for child and parent age, parent race/ethnicity, and parent education. All variables were coded such that higher ORs reflect a stronger intervention effect. Values <1 indicate a result in the opposite direction to that anticipated.

c 150 min/wk moderate-intensity or 75 min/wk vigorous-intensity aerobic PA (19).
